# Friedreich's Ataxia: Clinical Presentation of a Compound Heterozygote Child with a Rare Nonsense Mutation and Comparison with Previously Published Cases

**DOI:** 10.1155/2018/8587203

**Published:** 2018-08-09

**Authors:** Vamshi K. Rao, Christine J. DiDonato, Paul D. Larsen

**Affiliations:** ^1^Division of Neurology, Ann & Robert H. Lurie Children's Hospital of Chicago, Chicago, IL 60611, USA; ^2^Department of Pediatrics, Feinberg School of Medicine, Northwestern University, Chicago, IL 60611, USA; ^3^Human Molecular Genetics Program, Ann & Robert H. Lurie Children's Hospital, Stanley Manne Research Institute, Chicago, IL 60611, USA; ^4^Division of Neurology, Department of Pediatrics, University of Nebraska Medical Center and Children's Hospital and Medical Center, Omaha, NE, USA

## Abstract

Friedreich's ataxia is a neurodegenerative disorder associated with a GAA trinucleotide repeat expansion in intron 1 of the frataxin (FXN) gene. It is the most common autosomal recessive cerebellar ataxia, with a mean age of onset at 16 years. Nearly 95-98% of patients are homozygous for a 90-1300 GAA repeat expansion with only 2-5% demonstrating compound heterozygosity. Compound heterozygous individuals have a repeat expansion in one allele and a point mutation/deletion/insertion in the other. Compound heterozygosity and point mutations are very rare causes of Friedreich's ataxia and nonsense mutations are a further rarity among point mutations. We report a rare compound heterozygous Friedrich's ataxia patient who was found to have one expanded GAA FXN allele and a nonsense point mutation in the other. We summarize the four previously published cases of nonsense mutations and compare the phenotype to that of our patient. We compared clinical information from our patient with other nonsense FXN mutations reported in the literature. This nonsense mutation, to our knowledge, has only been described once previously; interestingly the individual was also of Cuban ancestry. A comparison with previously published cases of nonsense mutations demonstrates some common clinical characteristics.

## 1. Introduction

Friedrich's ataxia is the most common inherited ataxia with an estimated prevalence of 1 in 30,000-50,000 and a carrier frequency of 1 in 90-110 in the Caucasian population [1, 2]. It is characterized typically by progressive gait and limb ataxia, loss of deep tendon reflexes, and dysarthria. Additional features can include hypertrophic cardiomyopathy [3], diabetes [4], scoliosis, distal wasting, optic atrophy, and sensorineural deafness [5, 6].

The mutation causing Friedrich's ataxia was mapped to chromosome 9 by Chamberlain et al. in 1988 [7]. Subsequently in 1995 Montermini et al. [8] isolated the critical region on 9q13 and in 1996 Campuzano et al. [9] demonstrated the intronic GAA triplet repeat expansion that is associated with the disease.

The identification of the gene led to a phenotypic characterization of the disease. The classic phenotype (95-98%) associated with a homozygous GAA triplet repeat expansion (in the first intron) on both alleles has an age of onset before 25 years, wheelchair dependence within a decade, and death typically due to cardiac compromise by the fourth decade. Repeat expansion size was inversely correlated with the age at onset, duration to wheelchair, and development of cardiomyopathy [10]. Variability in clinical presentations included either late or very late presentations, retained tendon reflexes, or Acadian type (original French people living in North America, intermediate repeats, milder course, and lower incidence of cardiomyopathy) and was usually associated with lower GAA repeat expansion sizes or genetic modifiers.

Compound heterozygous (2-5%) individuals possess a GAA trinucleotide repeat expansion on one allele and a point mutation on the other allele. Diseases causing alterations include frameshift, missense, splice site, in/dels, and nonsense mutations. The two largest case series of Friedrich's ataxia patients with point mutations have been described by Cossee et al. [11] (25 patients) and Gellera et al. [12] (12 patients). Recently Galea et al. [13] compared clinical information from 131 individuals with homozygous expansions and 111 compound heterozygotes. Structural modeling and stability analyses were used to predict protein stability and protein interaction disruption of the various missense mutations. Within the 111 compound heterozygotes (81 were from previous literature review) 50 were predicted to be null alleles representing 38 different types of mutations. These consisted mostly of splice site or in/del mutations that resulted in truncating mutations. Very few were nonsense point mutations as they are rare among the point mutations that have been detected to date. It has been observed that null mutations have earlier onset and higher incidence of diabetes compared to homozygous GAA expansions. On the other hand, there is a higher rate of cardiomyopathy in homozygous GAA expansion than any type of compound heterozygous mutation.

Here we report the case of a child with Friedrich's ataxia found to harbor a W155X nonsense mutation that has to our knowledge, only been described once before [14]. We discuss the clinical phenotype while drawing comparisons with point mutations in general and the previously published cases of nonsense point mutations.

## 2. Case Description

A 7.5-year-old boy presented with progressive gait disturbance and falls. History included a full-term birth with no pregnancy or delivery complications. Developmental milestones including sitting up without support, walking, and speech were all within the normal range. Family history was remarkable for tremors in grandfather. He was first seen by the pediatric neurologist for unsteady gait and toe walking at the age of 3.5 years with the gait unsteadiness commencing around the age of 2.5 years with frequent falls. Tremors in the hands were noted sometime previous to the clinic visit. Examination was notable for a well-developed child with a normal funduscopic exam, no cardiac murmur, and normal mental status including speech, normal cranial nerves, and strength. He had 1+ deep tendon reflexes (DTRs) in both upper and lower extremities with down going toes. Gait was wide based and unsteady. He had a tremor in both hands.

By the age of 6.5 years he had progressed to more falls and worsening handwriting. Examination revealed pes cavus, mild scoliosis, and absence of cardiac murmur. Neurological exam was notable for trace to absent DTRs, loss of position sense, positive Romberg, downgoing toes, slowed rapid alternating movements, tremor on finger to nose exam, and wide based unsteady gait.

By the age of 7 years he had more frequent falls and worsening handwriting. Examination showed progression with respect to ataxia in upper and lower limbs with wider based gait. DTRs were absent and a positive Babinski was noted.

At last exam around the age of 7.5 years he was falling more, and exam showed evidence of increased tone in lower extremities with foot drop and steppage gait in addition to decreased proprioception in the lower extremities and inconsistent responses in the upper extremities.

Magnetic resonance imaging of the brain was normal. Laboratory testing including quantitative immunoglobulins, alpha fetoprotein, thyroid profile, serum lactate, vitamin E levels, creatine kinase, serum amino acids, and serum acylcarnitine profile were all normal. Echocardiogram showed global hypertrophy of both ventricles. Ophthalmological examination did not show any evidence of optic atrophy.

Mutation analysis showed one allele with a GAA trinucleotide repeat expansion of approximately 1000. Since the index of suspicion was high, frataxin sequencing was done which demonstrated another allele harboring a c.464G>A nucleotide change. The nucleotide change predicted an amino acid substitution of tryptophan to a premature stop codon at residue 155 (W155X).

## 3. Discussion 

**Figure 1:**
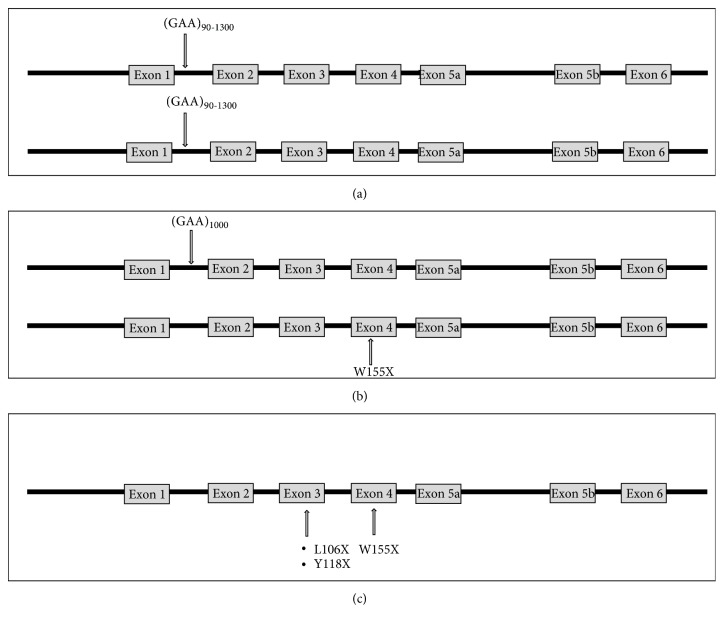
(a) Homozygous GAA triplet repeat expansion. (b) Compound heterozygosity with a point mutation in the second allele resulting in a stop codon, as in our patient. (c) Other known compound heterozygous cases resulting in a nonsense mutation from review of literature.

The classic Friedreich's ataxia phenotype (95-98%) is due to a homozygous GAA triplet repeat expansion in intron 1 of the FXN gene (Figure 1(a)), which results in low frataxin protein levels, whereas compound heterozygous (2-5%) individuals possess GAA trinucleotide repeat expansion on one allele and point mutation on the other allele, as seen in our patient (Figure 1(b)).

Including our patient there are four other reported nonsense mutations. Campuzano et al. [9] published the case of a French family with 2 affected siblings with a T to G transversion in exon 3 that changed a leucine to a stop codon (L106X). De Castro et al. [14] reported a Cuban patient from Florida, USA, with a G to A nucleotide change in exon 4 that resulted in substitution of tryptophan to a stop codon, similar to our patient (W155X). Gellera et al. [12] reported an Italian patient with a C to G transversion leading to a substitution of tyrosine to a stop codon (Y118X) in exon 3 (Figure 1(c)).

Clinical features of the known nonsense Friedrich's ataxia patients are listed in Table 1. All patients were males. Age at onset of symptoms was before 15. All patients presented with gait ataxia, upper motor neuron signs, dysarthria, decreased vibration, and scoliosis. Upper and lower limb areflexia and cardiomyopathy were present in all except sibling 2 from the French family. Diabetes was not found in any patients. Hearing loss was not present in most of the patients and presence or absence of hearing was not mentioned in the siblings from France.

From the collective works of Cossee et al. [11] and Gellera et al. [12], some patterns have emerged with respect to disease onset and clinical features when homozygous trinucleotide repeat expansion is compared to those with compound heterozygosity. As discussed in those papers, the onset of symptoms is significantly earlier in the compound heterozygous patients (before 25 years of age). The offered explanation is that the repeat expansion in the homozygous patients is significantly smaller and becomes the determinant of the age of onset. This seems to suggest that the residual expression of frataxin protein determines the clinical phenotype. Therefore, in compound heterozygotes the frataxin protein is very low in quantity with a larger repeat expansion on one allele and no protein is expressed with the point mutation on the other allele. Other clinical features that have been noted from the work of Cossee and Gellera et al. is the lower incidence of dysarthria and higher incidence of optic atrophy (especially where expansions were greater than 700 repeats).

Interestingly enough, all the patients with the known nonsense mutations were male. The nonsense mutation hot spots seem to center in exons 3 and 4 of the frataxin gene. Some features were consistent with what is known about compound heterozygotes with an early onset, upper motor signs, gait ataxia, decreased vibratory sensation, and cardiomyopathy. Age at onset was directly proportional to the number of trinucleotide repeat expansion sizes in the nonsense mutations too. Our patient demonstrated the earliest onset which correlated with approximately 1000 repeats, higher than the other patients. Our patient's ancestry on the paternal side was Cuban which was similar to the W155X nonsense mutation described by De Castro et al. [14].

The sample size for phenotypic generalization of children presenting with nonsense mutations is small and our patient is only 8 years of age. As such clinical pattern recognition with respect to absence of diabetes, hearing loss and optic atrophy in our patient cannot be thought of as unique. Furthermore, genetic and clinical heterogeneity has emerged within compound heterozygous mutations. Although lower levels of frataxin are seen in compound heterozygotes compared to homozygous GAA repeat expansions, levels of frataxin can be significantly different in tissues among different mutations, leading to variabilities in clinical phenotype [15]. There is evidence that certain mutations such as G130V or I154F, although compound heterozygote, have milder clinical phenotypes [16].

In conclusion, if the index of suspicion is high for Friedrich's ataxia then frataxin sequencing should be performed if there is a repeat expansion detected only on one allele. Secondly, for the most part, compound heterozygous patients have an earlier age of onset that directly correlates with the trinucleotide expansion size. Finally, whether there is a unique phenotype to the nonsense mutations requires further study before counseling families regarding natural history of disease.

## Figures and Tables

**Table 1:** Clinical features of Friedrich's ataxia patients with nonsense mutations.

Mutation	L106X (Sibling 1)	L106X (Sibling 2)	W155X	Y118X	W155X Our patient
Geographical origin	France	France	USA (Cuban origin)	Italy	USA (Father Cuban)

GAA repeat size	733	700	850	640	1000

Gender	Male	Male	Male	Male	Male

Age at onset (years)	9	13	4	14	2.5

Age at last exam (years)	35	32	Unknown	27	8

Gait ataxia	+	+	+	+	+

Nystagmus	+	+	-	+	-

Deep Tendon Reflexes	-	+, upper limbs	-	-	-

Babinski sign	+, unilateral	+	+	+	+

Vibration sense	↓	↓	↓	↓	↓

Foot deformity	+	+	+	-	+

Cardiomyopathy	+	-	+	+	+

Scoliosis	+	+	+	+	+

Optic disks	Pallor	Pallor	No atrophy	No atrophy	No atrophy

Dysarthria	+	+	+	+	+

Diabetes	-	-	-	-	-

Hearing loss	Not reported	Not reported	-	-	-
